# *In vitro* cytotoxicity of superheated steam hydrolyzed oligo((*R*)-3-hydroxybutyrate-*co*-(*R*)-3-hydroxyhexanoate) and characteristics of its blend with poly(L-lactic acid) for biomaterial applications

**DOI:** 10.1371/journal.pone.0199742

**Published:** 2018-06-26

**Authors:** Dhurga Devi Rajaratanam, Hidayah Ariffin, Mohd Ali Hassan, Nik Mohd Afizan Nik Abd Rahman, Haruo Nishida

**Affiliations:** 1 Department of Bioprocess Technology, Faculty of Biotechnology and Biomolecular Sciences, Universiti Putra Malaysia, UPM Serdang, Malaysia; 2 Graduate School of Life Science and Systems Engineering, Kyushu Institute of Technology, Hibikino, Wakamatsu, Kitakyushu, Fukuoka, Japan; 3 Institute of Tropical Forestry and Forest Products (INTROP), Universiti Putra Malaysia, UPM Serdang, Selangor, Malaysia; 4 Department of Cell and Molecular Biology, Faculty of Biotechnology and Biomolecular Sciences, Universiti Putra Malaysia, UPM Serdang, Malaysia; Osaka Shiritsu Daigaku, JAPAN

## Abstract

In order to clarify the *in vitro* cytotoxicity effect of superheated steam (SHS) treated poly((*R*)-3-hydroxybutyrate-*co*-(*R*)-3-hydroxyhexanoate) (PHBHHx) for biomaterial applications, SHS-treated PHBHHx oligoester samples: P(HB-*co*-6%-HHx) and P(HB-*co*-11%-HHx) with low and high percentages of unsaturated chain ends were evaluated for their cytotoxicity effects toward the growth of mouse fibroblast cell line NIH 3T3. From the results obtained after 24 and 48 h of the growth test, the SHS-treated PHBHHx oligoesters were found to be nontoxic to the growth of mouse fibroblast NIH 3T3 cell line with cell viability percentages of more than 95%. In order to serve as a potential resorbable medical suture, PHBHHx oligoesters were blended with poly(L-lactic acid) (PLLA) with a weight ratio of PHBHHx oligoester/PLLA = 20:80 (wt/wt) to improve mechanical properties of PHBHHx oligoesters. The PHBHHx oligoesters/PLLA blend films were evaluated for their thermal, mechanical, and surface wetting properties. Thermal properties of the blend films suggested a good compatibility between PHBHHx oligoesters and PLLA components. Mechanical properties of the blend films were determined to be close enough to a desirable strength range of medical sutures. Moreover, contact angle range of 65 < θ < 70° for the blend samples could provide desirable cell adhesion when used as biomaterials. Therefore, the blend of SHS-treated PHBHHx oligoesters and PLLA would be an ideal choice to be used as biomedical materials.

## Introduction

Polyhydroxyalkanoates (PHAs) are bacterial polymers produced as intracellular polymeric material through microbial fermentation under unbalanced nutrient conditions. Poly(3-hydroxybutyrate) (PHB), which is the most common representative of PHA family, exhibits a strong configuration of stereo regularity due to isotactic arrangements of pendant groups in its carbon backbone as a result of biologically catalyzed polymerization [1]. However, the configuration led to brittleness in its mechanical properties. Besides its brittleness, a narrow window processing ability makes it insufficient to replace the commercial plastics for packaging purposes [2].

Poly((*R*)-3-hydroxybutyrate-*co*-(*R*)-3-hydroxyhexanoate) (PHBHHx) is a third generation of PHA that consists of randomly distributed (*R*)-3-hyroxybutyrate (HB) and (*R*)-3-hydroxyhexanoate (HHx) units, has been improved with the mechanical properties and process ability compared to PHB and poly((*R*)-3-hydroxyvalerate) (PHBV) [3], resulting that PHBHHx is currently produced on a large scale. PHBHHx also has been proven to be biocompatible in clinical studies using mouse fibroblasts cells and rabbit articular cartilage-derived chondrocytes [4]. Biocompatibility is the most important factor for their practical tissue engineering applications besides their other favorable properties such as affinity towards various mammalian cells including smooth muscle cells, fibroblasts, chondrocytes, osteoblasts and bone marrow cells, *in-vitro* biodegradability as well as flexible mechanical properties [5,6].

Recent biocompatibility studies of PHAs have shown an immense degree of biocompatible and biodegradable properties, where the rate of biodegradation depended on the content of 3-hydroxyhexanoate (HHx) unit in PHBHHx [7]. On the other hand, *in vivo* studies of PHB and PHBV implants have been found to induce prolonged acute inflammatory responses [2, 8]. Despite the contribution of several factors to these phenomena, polymer surface was considered to be one of the most important factors due to its direct interaction with *in vivo* or *in vitro* environments. Poor polymer surface properties such as low surface free energy, smoothness and inert chemical compositions lead to reduced cell adhesions [6]. Thus, many studies have focused on the surface modifications such as alteration of chemical group functionality, topography, surface charge and surface wettability, which include its hydrophilic and hydrophobic properties [7,9]. Often, the aforementioned surface modifications were achieved through combination of chemical or physical means such as plasma treatment, electric discharge, surface grafting, surface hydrolysis, chemical reaction, metal vapor deposition, ultraviolet treatment as well as flame treatment [6,7,9,10] to serve the intended applications. Moreover, the degradation products of PHAs also influence the biocompatibility to some extent as previously reported for a degradation product of low molecular weight PHB, 3-hydroxybutyrate (3HB) [11], which naturally presents in human blood as a common metabolite [8]. Provided PHB and its oligomers and monomers are non-toxic to cells [10]. The derivatives of 3HB unit, which was found to increase the blood ketone bodies in mammals, reduce the cell line L929 death in high density cultures as well as shield against disease due to its therapeutic properties [11].

In our previous study, superheated steam (SHS) treatment was introduced for minor surface modification of the polymer that simultaneously reduced the molar mass of PHBHHx towards the production of oligoesters [12]. Previously, *in vitro* cytotoxicity effect was evaluated for oligo-hydroxyalkanoates (OHAs) with only saturated bonds in their chemical structure, where the lower concentration of OHAs (20 mg L^-1^) was found not to significantly affect cell viability of mouse fibroblast cell line L929 [11]. However, at higher temperatures, SHS treatment produced hydroxyl and alkenyl/unsaturated (crotonoyl and 2-hexenoyl) chain ends [12], where their *in vitro* cytotoxicity effect was unknown. Herein the present study proposed to produce biocompatible oligoesters that may serve as a biomaterial, due to the combined advantages of surface properties and the degradation products towards the biocompatibility.

In order to evaluate the biocompatibility of SHS hydrolyzed PHBHHx oligoesters using mouse fibroblast cell line NIH 3T3, two PHBHHx samples: P(HB-*co*-6%-HHx) and P(HB-*co*-11%-HHx) with low and high content of unsaturated chain ends as well as untreated PHBHHx samples as control were used. In general, the biocompatible PHBHHx appears to be a cell-friendly biomaterial for various biomedical applications as aforementioned. In order to propose a potential biomedical application for its oligoester, especially to serve as a resorbable surgical sutures having enough mechanical properties, it was blended with poly(L-lactic acid) (PLLA). PLLA represents another family of biodegradable polyester that also extensively studied for biomedical purposes due to its bioabsorbability [13]. To serve as surgical sutures, the intended polymeric material must exhibit exceptional tensile strength in order to effectively close the wound [14]. PHBHHx exhibits lower tensile strength with relatively higher percentage of elongation to break, contrastively PLLA exhibits high mechanical strength and good fabricability [13]. Therefore, by blending these two biodegradable polymers, the overall mechanical properties of the blend can be improved. The blends of PHBHHx oligomers and PLLA having good biocompatibility, fast degradability and improved mechanical properties would be a desirable polymeric materials to serve as resorbable surgical sutures for rapid-healing wounds. Moreover, this type of material can be used for other type of applications such as medical patch or artificial vascular grafts [15].

## Materials and methods

### Materials

PHBHHxs: P(HB-*co*-6%-HHx) (*M*_n_ 112,000, *M*_w_ 305,000 Da after purification) and P(HB-*co*-11%-HHx) (*M*_n_ 126,000, *M*_w_ 352,000 Da after purification) were kindly provided by Kaneka Corporation, Japan. Superheated steam (150 and 190°C) treated PHBHHxs: P(HB-*co-*6%-HHx) (SHS 150°C, *M*_n_: 31 kDa, *M*_w_: 73 kDa); P(HB*-co*-11%-HHx) (SHS 150°C, *M*_n_: 45 kDa, *M*_w_: 107 kDa); P(HB-*co-*6%-HHx) (SHS 190°C, *M*_n_: 2 kDa, *M*_w_: 4 kDa); P(HB*-co*-11%-HHx) (SHS 190°C, *M*_n_: 3 kDa, *M*_w_: 5 kDa) [12] were used for *in vitro* cytotoxicity evaluation. Superheated steam (170°C) treated PHBHHxs: P(HB-*co*-6%-HHx) (SHS 170°C, *M*_n_: 8 kDa, *M*_w_: 14 kDa); P(HB-*co*-11%-HHx) (SHS 170°C, *M*_n_: 10 kDa, *M*_w_: 21 kDa) [12] and poly(L-lactic acid) (PLLA) (U'z S-09, *M*_n_ of 66 kDa, *M*_w_ of 106 kDa, Toyota Eco Plastic, Japan) [16] was used for the preparation of PHBHHx/PLLA blend films. Chemicals and solvents: 3-(4,5-dimethylthiazol-2-yl)-2,5-diphenyl tetrazolium bromide (MTT) (Bio Basic Canada Inc, Canada), sodium bicarbonate (Sigma-Aldrich, USA), potassium chloride (Fisher Scientific, USA), potassium dihydrogen phosphate, anhydrous (Classic Chemicals, Malaysia), sodium phosphate dibasic anhydrous (Axil Scientific, Singapore), sodium chloride (Classic Chemicals, Malaysia), D(+)-glucose anhydrous (Classic Chemicals, Malaysia), trypLE^TM^ Express (Gibco Life Technologies, USA), Dulbecco’s modified eagle medium (Gibco Life Technologies, USA), fetal bovine serum (HyClone^TM^, Fisher Scientific, USA), hepes (Sigma-Aldrich, USA), dimethyl sulfoxide (DMSO) (Fisher Scientific, USA) and penicillin-streptomycin solution (Gibco Life Technologies, USA) were used as received.

### Cell culture of mouse fibroblast cell line NIH 3T3

The National Institute of Health (NIH) 3T3 cell line was obtained from American Type Culture Collection (ATCC, USA). The cell line was maintained in Dulbecco’s modified eagle medium (DMEM) supplemented with 10% fetal bovine serum (FBS) and 1% penicillin-streptomycin solution. The cells were grown in a humidified incubator supplemented with 5% CO_2_ at 37°C and cell morphological examinations of the culture flasks were performed daily under inverted light microscope (NIKON, Japan). After the cells were completely grown by covering the entire 75 cm^2^ culture flask (90% confluence), old media was discarded and the cells were washed with sterile phosphate buffer saline (PBS, pH 7.4) thrice, detached from the culture flasks using trypLE^TM^ Express (animal origin free) solution and passaged as single cells in a DMEM culture medium. The detached cells were collected, added with 10 mL of fresh DMEM medium to inactivate the action of trypLE^TM^ and centrifuged at 3000 rpm for 5 minutes to obtain the cell pellet. After centrifugation, supernatant was discarded, and 10 mL of fresh DMEM medium was added and was vortexed to obtain homogenous solution. The viable cells in the solution were counted by Trypan blue using hemocytometer.

### Cell viability and seeding of NIH 3T3

The viable cells were counted using Trypan blue cell count method in order to determine the seeding density of the NIH 3T3 cells. Approximately, a 10 μl of cell solution was added with 10 μl of 0.4% Trypan blue stain in a clean surface. Then, the cell suspension was immediately transferred to the edge of the hemocytometer chamber with a cover slip. The non-stained, shiny cells within four corners of grid were counted as viable cells under the inverted light microscope (NIKON, Japan). Based on the viable cell count obtained, serial dilution was performed to standardize the cell seeding density for each independent experiment. Five-hundred microliter of NIH 3T3 cells at a concentration of 6.25 x 10^4^ cells mL^-1^ was seeded into each well of 24-well tissue culture plate for 24 h. The following day, after initial morphological examination of NIH 3T3 cells under inverted light microscope, the untreated and SHS-treated PHBHHx films (P(HB-*co-*6%-HHx) and P(HB-*co-*11%-HHx)) with different weight contents (0.5, 1.0, 2.0, 4.0 and 8.0 mg) were prepared for the cytotoxicity evaluation and the cells in DMEM medium with the absence of PHBHHx films were served as control. Prior to inoculation, the films were sterilized under ultraviolet radiation for 10 min. After the PHBHHx films inoculation, the tissue culture plates were incubated for 24 and 48 h at 37°C under 5% CO_2_. The cell growth was observed by a phase-contrast microscopy (NIKON, Japan) and digital images were taken after 24 and 48 h inoculation.

### *In vitro* cytotoxicity assay on NIH 3T3 cells

The mouse fibroblast NIH 3T3 cell line was exposed to different weight contents (0.5, 1.0, 2.0, 4.0 and 8.0 mg) of PHBHHx films in the culture medium up to 24 and 48 h to determine the percentage of cell viability. After the incubation, PHBHHx films were removed and a total of 50μL of MTT solution (5mg mL^-1^ in PBS) was added into each well, continuing further incubation for 3 h at 37°C under 5% CO_2_ for formazan formation_._ Later, a 200μL of solution was removed from each well and a 200 μL of DMSO was added to dissolve the formazan crystal. Then, the absorbance readings of the wells on a plate were quantified at a wavelength of 550nm using ELISA microplate reader (Biotech Instruments, USA). The percentage of cell viability was calculated based on the absorbance readings which directly indicate the cell viability using Eq 1 [17]:

$$Cell\;viability(\%)=\frac{A_{film}}{A_{control}}\times 100$$ (1)

Where, *A*_film_ and *A*_control_ are absorbance values at 550nm of the cells with PHBHHx film and control, in which the cells where incubated in medium without PHBHHx film, respectively. Each experiment was conducted in triplicate.

### Preparation of SHS-treated PHBHHx/PLLA blends

SHS-treated PHBHHx/PLLA blends were prepared by melt mixing with the ratio of PHBHHx/PLLA = 20/80 (wt/wt) using a twin-screw extruder (IMC-1979, Imoto Machinery Co., Ltd., Japan) at 180°C for 10 min at rotation speed of 50 rpm. Resulting melt-compounded blends were cut into smaller pieces. Blend films were prepared using a compressed molding technique with heat pressure film forming machine IMC-180C (Imoto Machinery Co., Ltd., Japan) at 180°C under pressure of 50 MPa for 10 min and followed by cooling to room temperature. Thickness of the transparent films formed was about 0.2 mm.

### Characterization

Melting behavior and miscibility of neat PLLA and PHBHHx/PLLA (20/80 wt/wt) blend films were determined using a differential scanning calorimetry (DSC Q20, TA Instruments, USA). Samples were heated in a temperature range of 30–200°C at a heating rate of 10°C min^-1^ under nitrogen atmosphere. Melting point (*T*_*m*_) was taken from the highest point of melting peak.

Wide angle X-ray diffraction (WAXD) analysis was performed on an X-Ray Diffractometer RINT 2100 (Rigaku Corporation, Tokyo, Japan), operated at 40 kV and 20 mA with Cu Kα (λ = 0.1542 nm) radiation. Every scan was recorded in a range of 2θ = 5–50° under room temperature at a scan speed of 10° min^-1^. Crystallinity value: *X*_*c*_ (%) was determined by calculating the ratio of crystalline area to total area of profile in diffractogram (Eq 2), where the scattering profile of the amorphous area was taken into account.

$$X_{c}\;\left(\%\right)=\frac{Area\;of\;peaks\;in\;crystal\;phase}{Total\;area\;of\;all\;peaks}\times 100$$ (2)

Measurements of mechanical properties of rectangular shape films (40 x 5 x 0.2 mm) were performed on a tensile analyzer IMC-18E0 (Imoto Machinery Co., Ltd., Japan). The mechanical properties of films were measured by a stretching mode at a cross-head speed of 5 mm min^-1^ up to their breaking points at 25°C. The analysis was done in triplicate.

Hydrophilic properties of the samples were measured on a water contact angle equipment (Biolin Scientific, Finland) packaged with OneAttension software using a sessile drop method. A drop of 3.0 μL of distilled water was gently dropped onto the surface of PHBHHx, PLLA and PHBHHx/PLLA blend films and its contact angle value (θ) was estimated as a mean of values recorded after 10 s. The analysis was repeated thrice at random locations of the film surface.

## Statistical analysis

The experimental data were expressed as the mean ± standard deviation. The analysis was performed with one-way analysis of variance (ANOVA) and the group means were compared by Duncan’s multiple range test using SPSS software version 16. Values of *p* < 0.05 were considered as statistically significant.

## Results and discussion

### MTT cell viability of NIH 3T3 cells

The *in vitro* cytotoxicity of SHS-treated PHBHHx samples was determined for 24 and 48 h using mouse fibroblast cell line NIH 3T3 for PHBHHxs: P(HB-*co*-6%-HHx) and P(HB-*co*-11%-HHx), with low and high content of unsaturated chain ends, where the untreated PHBHHxs were used as control. The percentage of unsaturated chain ends of SHS-treated PHBHHx films at 150 and 190°C were previously reported by Rajaratanam et al. [12] (S1 Table). Desirable biomaterials should be biocompatible and cause no adverse effect to surrounding tissues. Thus, the *in vitro* cytotoxicity assay (MTT assay) was performed to predict the biocompatibility of SHS-treated PHBHHx samples with their corresponding percentage of chain ends for the *in vivo* applications. In many previous cell viability studies, the PHBHHx films were found to be biocompatible, non-toxic and even tremendously increased the cell viability and cell proliferation rate of the tested cell lines. For example, Yang et al. [9] reported that growth of mouse fibroblast cell line L929 on PHBHHx film was 216 and 11 times better than those on PHB and PLA films, respectively. They also found that as the percentage of PHBHHx content increased in the blend with PHB, the number of cell growth on the polymer surface also greatly increased. Later, Sun et al. [11] examined the cell viability in the presence of medium chain length oligo(3-hydroxyalkanoates) particles using mouse fibroblast cell line L929 and found that a concentration range of 2–200 mg L^-1^ did not significantly affect the growth of the cell lines and the cytotoxicity effect of OHAs decreased with increasing side chain content of OHAs such as PHBHHx. Despite the proven data of PHBHHx biocompatibility, Sun et al. [11] reported that the presence of unsaturated bonds in the chemical structure of polymer surface might be toxic for the growth of cells. However, there was no proven data to support the claim to date. As reported in the previous paper [12], the SHS-treated PHBHHx samples had unsaturated chain ends. Therefore, to ensure the biocompatibility of the samples having unsaturated chain ends, the cell viability assay was conducted.

**Fig 1:**
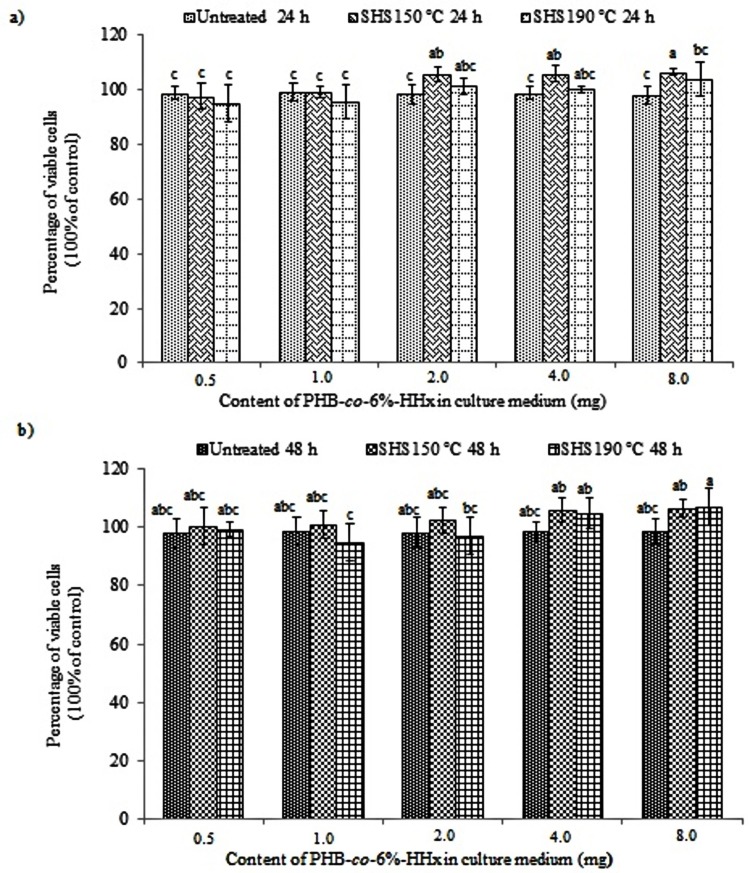
Cell viability of NIH 3T3 cells incubated with untreated and SHS-treated P(HB-*co*-6%-HHx) samples. (a) Cell cultured for 24 and (b) 48 h. The mean data denoted by different superscript letters are significantly different (Duncan’s multiple range test, *p* < 0.05).

**Fig 2:**
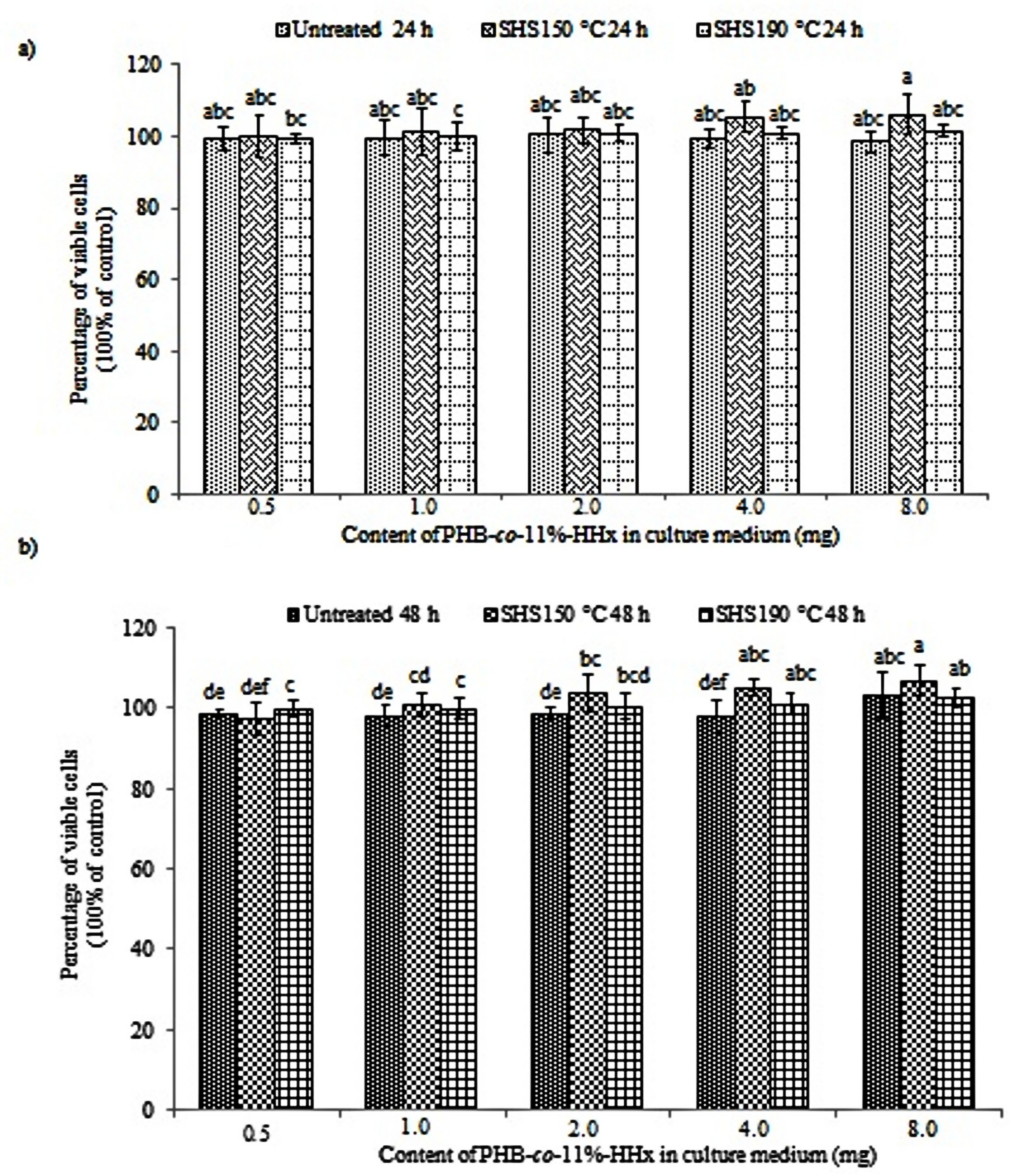
Cell viability of NIH 3T3 cells incubated with untreated and SHS-treated P(HB-*co*-11%-HHx) samples. (a) Cell cultured for 24 and (b) 48 h. The mean data denoted by different superscript letters are significantly different (Duncan’s multiple range test, *p* < 0.05).

DMEM medium was used to culture the mouse fibroblast NIH 3T3 cells as it has been proven to increase the media stability and cell performance by reducing the toxic ammonia built up [18]. After 24 and 48 h, MTT assay was performed in order to quantitatively evaluate the cell viability. In general, the MTT assay involves the conversion of tetrazolium bromide salt, 3-(4,5-dimethylthiazol-2-yl)-2,5 diphenyl tetrazolium bromide into an insoluble formazan crystals, which is a purple colored compound in mitochondria of living cells and can be quantified by spectrophotometric analysis [9]. The percentages of cell viability of NIH 3T3 cells with untreated and SHS-treated PHBHHx films with different weights were shown in Figs 1 and 2.

As shown in Figs 1 and 2, the NIH 3T3 cells were viable up to 48 h in the presence of untreated and SHS-treated PHBHHx samples with low and high percentages of unsaturated chain ends (crotonoyl and 2-hexenoyl). As listed in S1 Table, the low percentage of unsaturated chain ends were obtained at SHS treatment temperature of 150°C with 31.9% for P(HB-*co-*6%-HHx) and nearly no detectable unsaturated chain ends for P(HB-*co*-11%-HHx). However, at SHS treatment temperature of 190°C, high percentage of unsaturated chain ends, 80.8 and 81.7% were obtained for P(HB-*co*-6%-HHx) and P(HB-*co-*11%-HHx), respectively. The typical spindle morphology of the mouse fibroblast cell line NIH 3T3 was remained unchanged during the incubation periods of 24 and 48 h in the presence of PHBHHx samples having different percentages of unsaturated chain ends. The morphology of cell line shown in S1 Fig was compared with representative SHS-treated PHBHHx samples at 150°C. The percentages of viable cell were found to be almost the same with the control or at least above 95%. This is an interesting finding as it shows the positive effect of SHS treatment toward the cell viability of mouse fibroblast cell line up to 48 h, due to the fact that SHS hydrolysis able to produce biocompatible PHBHHx oligoester for further biomaterial applications. Previously, ISO 10993–5 (2009) reported that if the percentage of cell viability exceeds 80%, it is considered to be non-toxic [17]. Therefore, it can be concluded that the SHS-treated PHBHHx samples with unsaturated crotonoyl and 2-hexenoyl chain ends possess no cytotoxicity effect and can be used as biomaterials. Moreover, the different weights of samples also insignificantly affected the cell proliferation rate of the NIH 3T3 cells.

### Characterization of oligo(HBHHx)/PLLA blends

As discussed above, the SHS-treated PHBHHx samples were found to be non-cytotoxic and ideal for the development of functional biomaterials, preferably for resorbable surgical sutures. The most important properties of the ideal resorbable sutures are high biocompatibility, smooth surface that prevents injuries on surrounding tissues, easy to handle and knot, high tensile strength to close the wounds and absorption into body after the serving duration [15]. Generally, since fast-degrading sutures are required for fast-healing tissues, the usage of PHBHHx oligoester with lower molecular weight aids the fast degradation process of sutures *in vivo*. However, the PHBHHx oligoester is solely not enough for the sutures because they require high tensile strength and better elongation to break than properties of PHBHHx oligoester itself, although high molecular weight PHBHHx naturally exhibits exceptionally high elongation to break and contrastively lower tensile strength [13], suggesting easy to produce thin fibers with uniform texture.

Therefore, the incorporation of second polymer such as PLLA into PHBHHx oligoester may be able to overcome the problem [15]. Since PLLA exhibits high mechanical strength, the blend of PLLA and PHBHHx oligoester may be able to improve the overall mechanical properties. Thus, in the present study, PHBHHx oligoesters were blended with PLLA in the weight ratio of PHBHHx oligoesters/PLLA = 20:80 (wt/wt) which ratio was found to remarkably improve the mechanical properties of blends by increasing the toughness [13]. Moreover, Zhao et al. [13] reported that PHBHHx/PLLA (20/80 wt/wt) with *M*_w_ of 624 and 192 kDa, respectively, showed higher degree of compatibility with homogenous one-phase pattern based on Fourier transform infrared (FTIR)-microscopic analysis. In order to prepare the PHBHHx oligoester/PLLA (20/80 wt/wt) blend, two kinds of PHBHHx oligoesters produced by SHS-treatment at 170°C: (P(HB-*co*-6%-HHx), *M*_w_ 14 kDa and P(HB-*co*-11%-HHx), *M*_w_ 21 kDa which were in the preferable molecular weight range of PHA oligomers: 1,200 to 25,000 Da [19], were used. For the discussion purpose in the following sections, the SHS-treated PHBHHx samples at 170°C, were simply expressed as oligo(HBHHx): oligo(HB-*co*-6%-HHx) and oligo(HB-*co*-11%-HHx). Besides having molecular weight in the desirable range, oligo(HBHHx) also had a medium percentage of unsaturated chain ends, 68.9 and 62.1% for oligo(HB-*co*-6%-HHx) and oligo(HB-*co*-11%-HHx), respectively. Since the high percentage of unsaturated chain ends found to be nontoxic as abovementioned, oligo(HBHHx) with medium percentage of unsaturated chain ends is desirable to be blended with PLLA for its application as a biomaterial.

### Thermal properties of oligo(HBHHx)/PLLA blends

**Fig 3:**
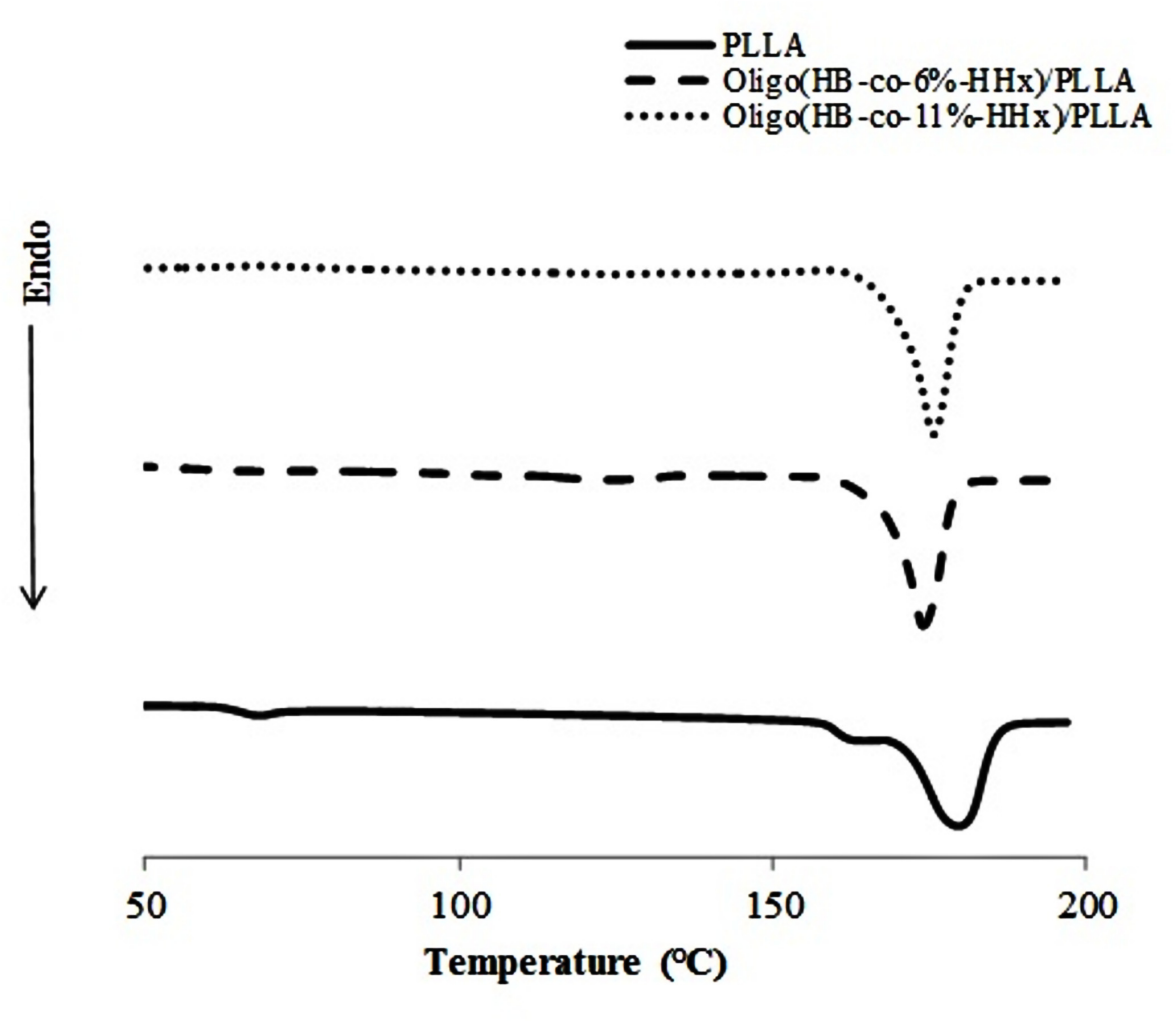
DSC thermograms of neat PLLA and oligo(HBHHx)/PLLA blends.

The thermal properties of the oligo(HBHHx)/PLLA blends were obtained from the heating scan of DSC (Fig 3 and Table 1). As previously discussed [12], the untreated and SHS-treated PHBHHx samples had multiple melting peaks. However, after the addition of PLLA, the multiple melting peaks are not detected, which could be mainly due to suppression effect of the crystallization of PHBHHx by PLLA. Moreover, although the neat PLLA showed a glass transition temperature (*T*_g_) of 67.4°C, interestingly, the *T*_g_ of neat PLLA was not detected in oligo(HBHHx)/PLLA blends. This suggests that the *T*_g_ that mainly depends on the amorphous phase of the material, might have shifted to a lower temperature range. This shift is possible and acceptable because in a previous report [13] phase morphology of a blend of PHBHHx/PLLA (20/80 wt/wt) resulted in one-phase as a compatible blend, leading to the shift of *T*_g_ to a lower temperature region. The lower *T*_g_ value of the blends also may be advantageous for further manufacturing in a larger scale.

**Table 1:** Thermal and physical properties of neat PLLA and oligo(HBHHx)/PLLA blends.

Sample	*T*_m_^a^ (°C)	Δ*H*_m_^a^ (J/g)	*T*_g_^a^ (°C)	*X*_*c*_^*b*^(%)
Neat PLLA	179.6	54.1	67.4	70.0
Oligo(HB-*co*-6%-HHx)	123.8, 138.7	64.4	n.d	43.9
Oligo(HB-*co*-11%-HHx)	95.2, 125.1, 144.1	41.5	n.d	28.3
Oligo(HB-*co*-6%-HHx)/PLLA	174.0	48.4	n.d	52.5
Oligo(HB-*co*-11%-HHx)/PLLA	175.6	48.2	n.d	39.9

^a^determined by DSC.

^b^determined by WAXD.

n.d—not detected in a temperature range of 30–200°C.

Moreover, there were observable changes in enthalpy of melting (Δ*H*_m_) of oligo(HBHHx)/PLLA blends, which were close to Δ*H*_m_ of neat PLLA, however, lower than oligo(HB-*co-*6%-HHx) and higher than oligo(HB-*co-*11%-HHx). Therefore, this suggests that PLLA acts as partially compatibilizing agent with the crystal phase of PHBHHx and fully compatible with the amorphous region of PHBHHx. This can be further supported by results of crystallinity (*X*_c_) obtained by WAXD analysis (Table 1). As listed in Table 1, *X*_c_ values of neat PLLA, oligo(HB-*co*-6%-HHx)/PLLA and oligo(HB-*co*-11%-HHx)/PLLA were 70.0, 52.5, and 39.9%, respectively. The *X*_c_ values of original oligo(HB-*co*-6%-HHx) and oligo(HB-*co*-11%-HHx) were 43.9 and 28.3%, respectively (S2 Table). Therefore, it is evident that the addition of PLLA not only affects the amorphous phase, but also the crystalline phase of PHBHHx significantly.

### Mechanical properties of oligo(HBHHx)/PLLA blends

**Fig 4:**
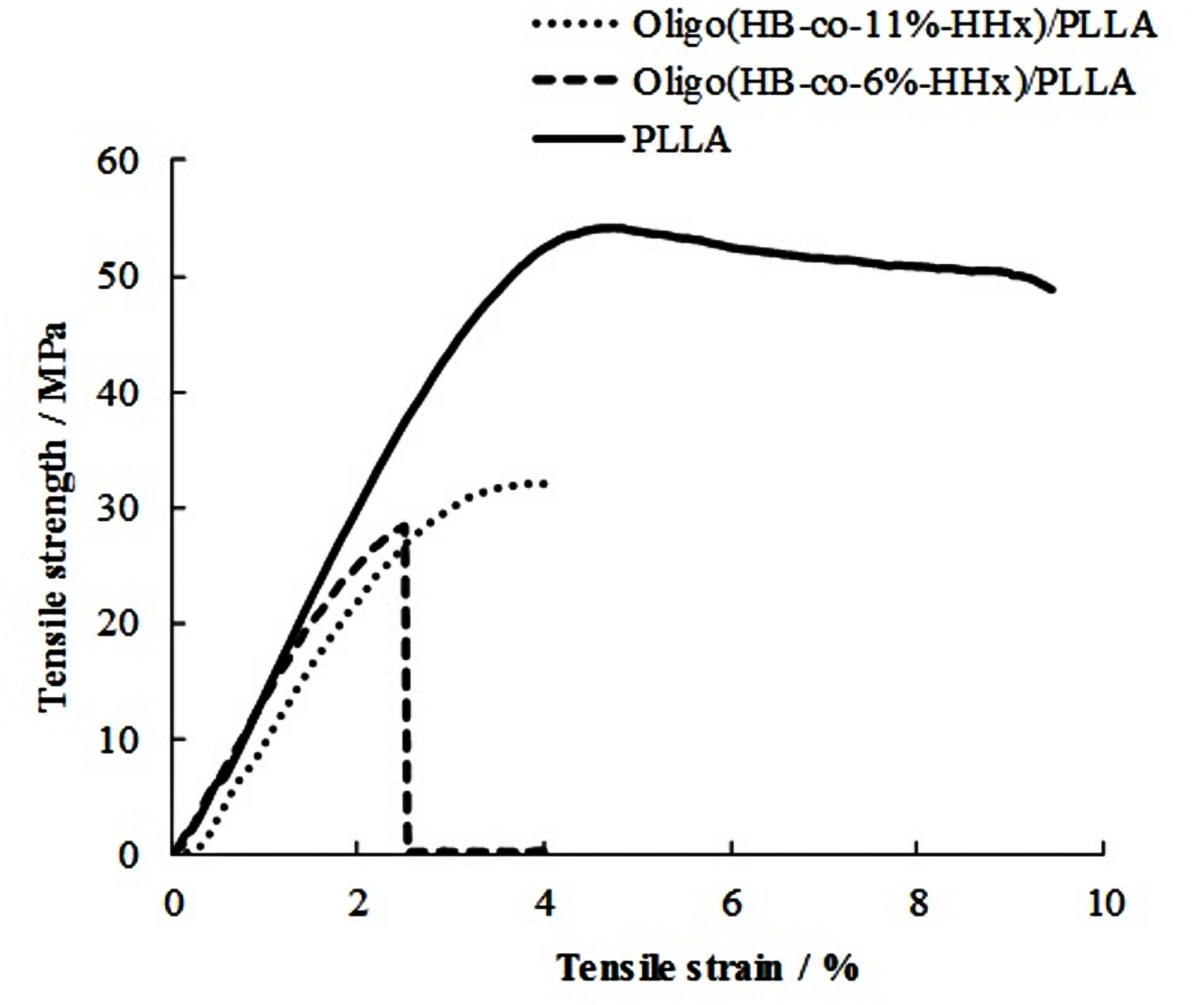
Stress-strain curves for neat PLLA and oligo(HBHHx)/PLLA blends.

Mechanical properties are important characteristics for medical sutures. The desirable ranges of Young’s modulus, tensile strength, and elongation at break (%) for medical sutures were ~850 MPa, 37±10 MPa, and ~70%, respectively [20]. As listed in Table 2, it was found that tensile strength values of neat PLLA, oligo(HB-*co*-6%-HHx)/PLLA and oligo(HB-*co*-11%-HHx)/PLLA were 54.2, 28.6, and 33.8 MPa, respectively, where the stress-strain curves are shown in Fig 4. From results in Fig 4, oligo(HB-*co*-6%-HHx)/PLLA showed a brittle fracture, meanwhile oligo(HB-*co*-11%-HHx)/PLLA, which had higher percentage of 3HHx unit in the backbone structure of PHBHHx showed improvement in the ductility. This observation can be explained by the presence of higher portion of bulky side group (3HHx), which side group interfered the polymer chain arrangement, caused lower crystallinity (Table 1) and hence, resulted in a more ductile copolymer. It is interesting to note that PLLA, which had the highest crystallinity, showed a partially ductile fracture as observed in Fig 4. Despite the fact that PHBHHx is a more ductile polymer than PLLA due to its low *T*_g_ [13], oligoesters have many chain ends as defects in the mechanical properties. Moreover, the lamella structure of PHBHHx might be more developed crystalline in size, whereas small sized PLLA crystalline–rich region could provide higher resistance towards tilting that led to the fracture or deformation of blends as shown in Fig 4.

The mechanical properties of oligo(HB-*co*-11%-HHx)/PLLA are close to the desired ranges of medical sutures except the elongation at break. The results show that as the percentage of HHx unit increased, the tensile strength was increased as well as the elongation. PHBHHx is generally regarded as a type of soft and flexible polyester with low tensile strength and Young’s modulus, however possesses good elongation at break. Previously, [13] reported that the PHB-*co*-20%-HHx/PLLA (20/80 wt/wt, *M*_w_ 624 kDa) blend exhibited similar mechanical properties except the elongation at break as listed in Table 2.

**Table 2:** Mechanical properties of neat PLLA and oligo(HBHHx)/PLLA blends.

Samples	Tensile strength (MPa)	Young’s modulus (GPa)	Elongation at break (%)	References
Neat PLLA (Toyota Eco Plastic)	54.2 ± 0.0	1.7 ± 0.0	5.6 ± 3.3	This study
Oligo(HB-*co*-6%-HHx)/PLLA	28.6 ± 1.2	1.4 ± 0.0	2.6 ± 1.3	This study
Oligo(HB-*co*-11%-HHx)/PLLA	33.8 ± 2.0	1.2 ± 0.1	3.6 ± 0.7	This study
Neat PLLA (Shimadzu)	36.4 ± 3.9	1.4 ± 0.1	13.8 ± 5.7	[13]
(PHB-*co*-20%-HHx)/PLLA (20/80)	29.5 ± 0.9	1.3 ± 0.0	99.6 ± 69.4	[13]

The elongation at break (%) of the blends in the present study was much lower, which can be explained by the differences not only in molecular weight, but also in % of HHx unit in PHBHHx samples. In the previous study, PHBHHx with 20% 3-HHx unit was used [13], while in this study PHBHHxs with 6 and 11% 3-HHx unit were used. It has been reported that increasing the content of 3-HHx unit leads to a more flexible polymer [21]. Therefore, it can be concluded that the addition of PLLA into PHBHHx oligoesters resulted in the improvement in mechanical properties due to the interaction not only with amorphous phase, but also partially with crystalline phase of PHBHHx, caused by the fine distribution of the small sized stiff PLLA molecules into the continuous phase of PHBHHx matrix.

### Surface wettability of oligo(HBHHx)/PLLA blends

**Fig 5:**
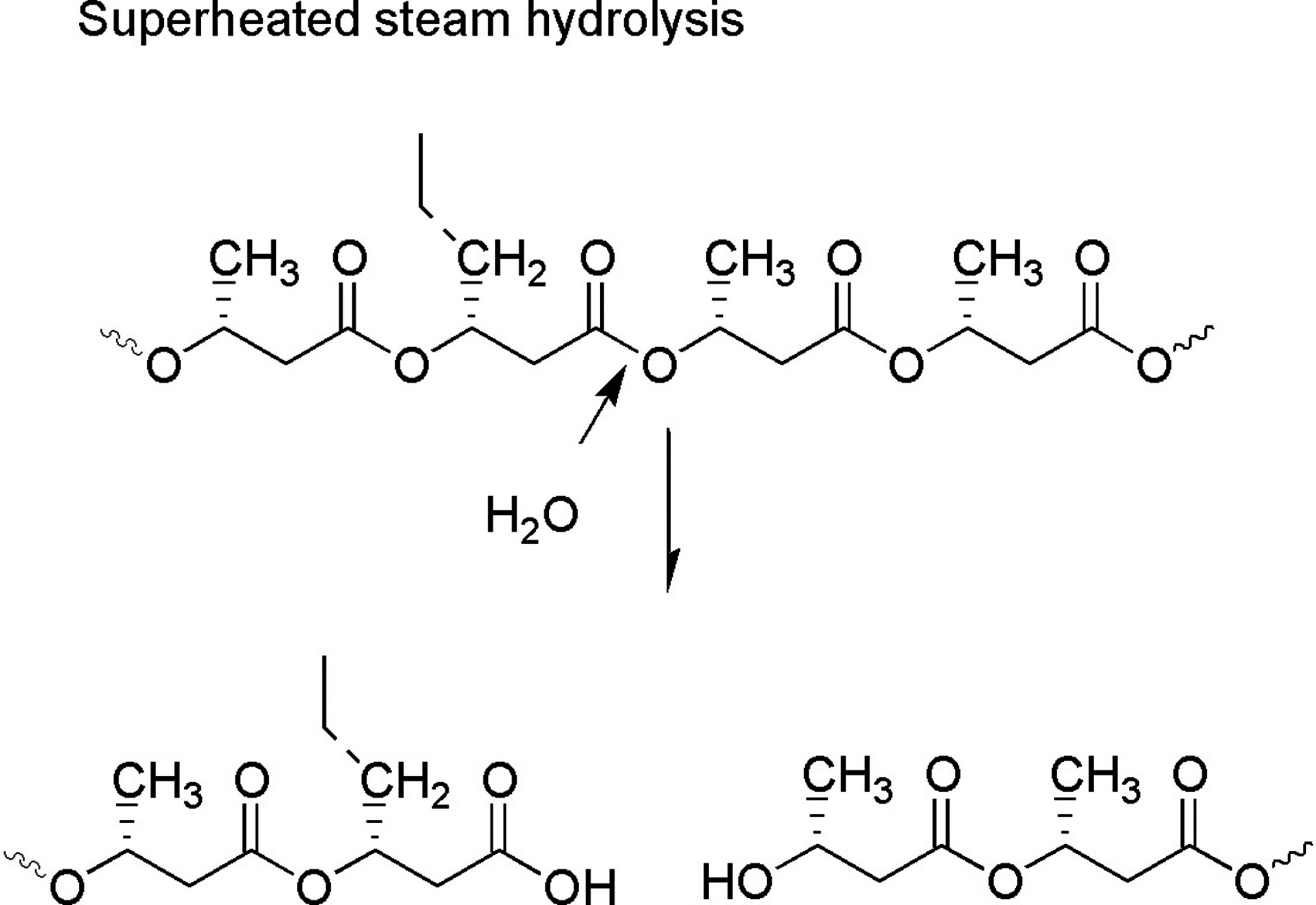
Oligoesters with hydroxyl chain-end.

Surface wettability of PLLA, neat PHBHHx, SHS-treated PHBHHx and oligo(HBHHx)/PLLA blends were determined by measurement of contact angle. The characteristics of surface wettability would provide the information on hydrophilic characteristics of polymer surface, which is important factor for the cell attachment and cell proliferation on surface. In Table 3, the contact angle values of neat polymers and blend samples are shown. In general, the surface with contact angle value of 0 < θ < 90° is regarded as good wetting with hydrophilicity, and the surface with contact angle value of 180 > θ > 90° is regarded as incomplete or no wetting due to its hydrophobicity. Therefore, both neat polymer and blend samples were regarded as slightly hydrophilic materials, because the contact angle values were lower than 90°. Each oligo(HBHHx) exhibited lower contact angle value than corresponding neat PHBHHx. This could be due to the higher contents of hydrophilic hydroxyl chain ends of oligo(HBHHx)s (Fig 5). The hydroxyl groups interact with water molecules to form hydrogen bonds that lead to decrease in the interfacial free energy of oligoester surfaces, resulting in increases of the overall surface hydrophilicity. Small increases in contact angle value of blends were noticed compared to neat oligoesters. It could be results of blend with PLLA.

**Table 3:** Contact angle values of PLLA, PHBHHx and oligo(HBHHx)/PLLA blends.

Samples	Contact angle (°)
Neat PLLA	67.1 ± 0.5
Neat P(HB-*co*-6%-HHx)	73.3 ± 1.9
Neat P(HB-*co*-11%-HHx)	77.5 ± 1.3
Oligo(HB-*co*-6%-HHx)	64.8 ± 1.2
Oligo(HB-*co*-11%-HHx)	65.5 ± 1.9
Oligo(HB-*co*-6%-HHx)/PLLA	67.8 ± 0.9
Oligo(HB-*co*-11%-HHx)/PLLA	68.5 ± 1.7

In general, surfaces that are too hydrophilic or too hydrophobic have been reported to be non-optimal for cell adhesion. Wang et al. [22] reported that among PHBHHx samples with HHx content in a range of 5 to 20%, the PHBHHx with 20% HHx showed a contact angle of 85°, and the highest cell density of fibroblast proliferation and the PHBHHx with 12% HHx showed the highest cell density of osteoblast proliferation. Later, Xu and Siedlecki [23] reported that low density polyethylene (LDPE) surfaces with contact angle values in the range of 60–65° showed stronger protein adhesion of bovine serum albumin, fibrinogen and human FXII than surfaces with θ < 60°. On the other hand, chinese hamster ovary (CHO), fibroblasts, and endothelial cells showed the maximum adhesion on LDPE surface with contact angle of 55° [24]. Tamada and Ikada [25] reported that the optimal water contact angle value of polymers for the mice fibroblast cell adhesion was around 70°. As reported by such previous studies, the cell adhesion varies for different polymer surfaces and cell lines. Generally, the preferable water contact angle values for cell adhesion were found to be in the range of 55–85° as abovementioned. Therefore, the contact angle range of 65 < θ < 70° obtained in the present study for PHBHHx oligoesters and oligo(HBHHx)/PLLA blend samples could provide desirable cell adhesion when used as a biomaterial.

## Conclusions

The present study evaluated the *in vitro* cytotoxicity effect of superheated steam treated PHBHHx samples for the development of functional biomaterial, preferably resorbable medical sutures. Untreated and SHS treated (150 and 190°C) PHBHHx samples with low and high percentage of unsaturated chain ends were found to be non-cytotoxic for the growth of mouse fibroblast cell line NIH 3T3 with cell viability percentage of more than 95%. Since, the SHS hydrolyzed PHBHHx oligoesters were found to exhibit high biocompatibility, it was further evaluated to be potentially used as resorbable medical sutures. Natural biodegradable polyester PLLA was used to blend with PHBHHx matrix with the weight ratio of (80/20) in order to improve mechanical properties of the films. The combined effect of increased toughening and reinforcing effect of PLLA, and the faster degradation rate resulted from low molecular weight of PHBHHx oligoesters, would be an ideal choice for the fabrication of resorbable medical sutures that requires rapid-healing.

## Supporting information

S1 Fig Cell morphology of NIH 3T3 cell line. (a) Control cell line (b) SHS treated P(HB-*co*-6%-HHx) at 150°C (8mg) (c) SHS treated P(HB-*co*-11%-HHx) at 150°C (8mg). Solid arrows indicate the morphology of mouse fibroblast NIH 3T3 cell lines.
(DOCX)

S1 Table Quantitative analytical results of chain-end structures of PHBHHxs samples after SHS treatment [12]. (DOCX)

S2 Table Thermal properties of SHS treated PHBHHx samples based on DSC thermograms for treatment temperature of 170°C. (DOCX)
