# Supplementary material for: In vitro cytotoxicity of superheated steam hydrolyzed oligo((R)-3-hydroxybutyrate-co-(R)-3-hydroxyhexanoate) and characteristics of its blend with poly(L-lactic acid) for biomaterial applications
Source: PLoS One. 2018 Jun 26;13(6):e0199742. doi: 10.1371/journal.pone.0199742 (PMC6019698; doi:10.1371/journal.pone.0199742)
Supplement: S1 Fig — (a) Control cell line (b) SHS treated P(HB-co-6%-HHx) at 150°C (8mg) (c) SHS treated P(HB-co-11%-HHx) at 150°C (8mg). Solid arrows indicate the morphology of mouse fibroblast NIH 3T3 cell lines. (DOCX) [file pone.0199742.s001.docx]

**Supporting information**


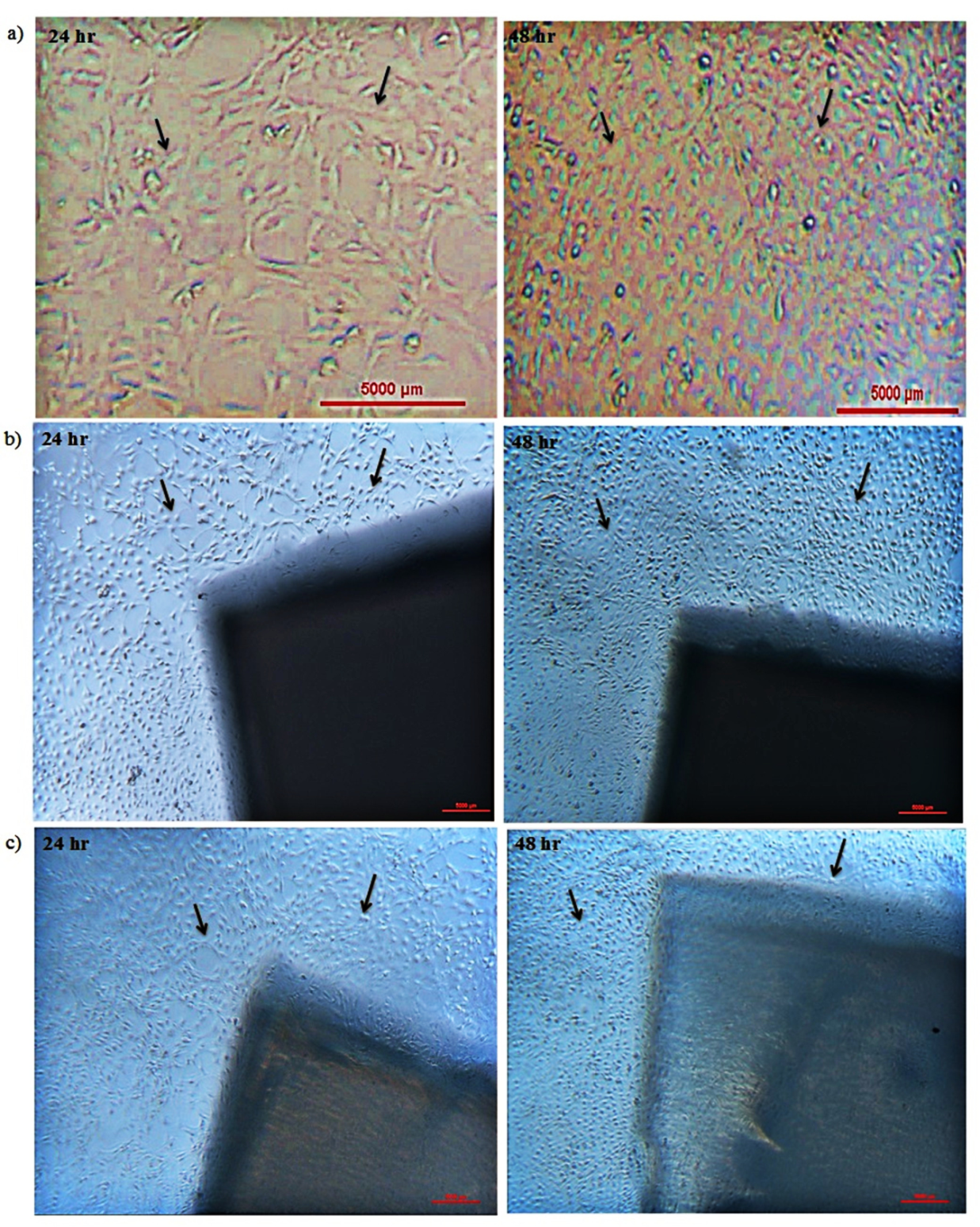


**S1 Fig. Cell morphology of NIH 3T3 cell line** (a) Control cell line (b) SHS treated P(HB-*co*-6%-HHx) at 150 °C (8mg) (c) SHS treated P(HB-*co*-11%-HHx) at 150 °C (8mg). Solid arrows indicate the morphology of mouse fibroblast NIH 3T3 cell lines.
