# Supplementary material for: In vitro cytotoxicity of superheated steam hydrolyzed oligo((R)-3-hydroxybutyrate-co-(R)-3-hydroxyhexanoate) and characteristics of its blend with poly(L-lactic acid) for biomaterial applications
Source: PLoS One. 2018 Jun 26;13(6):e0199742. doi: 10.1371/journal.pone.0199742 (PMC6019698; doi:10.1371/journal.pone.0199742)
Supplement: S1 Table — (DOCX) [file pone.0199742.s002.docx]

**Supporting information**

**S1 Table. Quantitative analytical results of chain-end structures of PHBHHx samples after SHS treatment [12].**

| Sample | Treatment  condition | | Percentage (%)  of chain-end groups | | |
| --- | --- | --- | --- | --- | --- |
|  | **Temperature**  **(°C)** | **Time (min)** | **3-hydroxy butanoyl**  **(HB unit)** | **Crotonoyl**  **(HB unit)** | **2-hexenoyl**  **(HHx unit)** |
| P(HB-*co-*6%-HHx) | 130 | 600 | 100.0 | n.d* | n.d |
|  | 150 | 400 | 68.1 | 31.9 | n.d |
|  | 170 | 300 | 31.1 | 68.9 | n.d |
|  | 190 | 200 | 19.2 | 76.5 | 4.3 |
| P(HB-*co*-11%-HHx) | 130 | 600 | 100.0 | n.d | n.d |
|  | 150 | 400 | 100.0 | n.d | n.d |
|  | 170 | 300 | 37.9 | 62.1 | n.d |
|  | 190 | 200 | 18.3 | 74.5 | 7.2 |

^*^ Undetected.
