# Supplementary material for: In vitro cytotoxicity of superheated steam hydrolyzed oligo((R)-3-hydroxybutyrate-co-(R)-3-hydroxyhexanoate) and characteristics of its blend with poly(L-lactic acid) for biomaterial applications
Source: PLoS One. 2018 Jun 26;13(6):e0199742. doi: 10.1371/journal.pone.0199742 (PMC6019698; doi:10.1371/journal.pone.0199742)
Supplement: S2 Table — (DOCX) [file pone.0199742.s003.docx]

**Supporting information**

**S2 Table. Thermal properties of SHS treated PHBHHx samples based on DSC thermograms for treatment temperature of 170 °C.**

| PHA sample | SHS treatment | | *T*_m_  (°C) | ∆*H*_m_  (J/g) | *X*_c_  (%) |
| --- | --- | --- | --- | --- | --- |
|  | **Temperature (°C)** | **Time**  **(min)** |  |  |  |
| PHB-*co*-6%-HHx | 170 | 30 | 121.7, 142.1 | 59.2 | 40.4 |
|  |  | 300 | 123.8, 138.7 | 64.4 | 43.9 |
| PHB-*co*-11%-HHx | 170 | 30 | 100,7, 122.9 | 40.9 | 27.9 |
|  |  | 300 | 95.2, 125.1, 144.1 | 41.5 | 28.3 |
